# Corticosteroid and antimicrobial therapy in macrolide-resistant pneumococcal pneumonia porcine model

**DOI:** 10.1186/s40635-025-00731-1

**Published:** 2025-02-27

**Authors:** Ana Motos, Minlan Yang, Denise Battaglini, Hua Yang, Andrea Meli, Joaquim Bobi, Roberto Cabrera, Giacomo Tanzella, Carmen Rosa Vargas, Marta Arrieta, Blanca Llonch, Nona Rovira-Ribalta, Enric Barbeta, Pierluigi di Giannatale, Stefano Nogas, Laia Fernández-Barat, Montserrat Rigol, Kasra Kiarostami, Ignacio Martín-Loeches, Jordi Vila, Daniel Martinez, Gianluigi Li Bassi, Antoni Torres

**Affiliations:** 1 Pneumology Department, Hospital Clínic, Thorax Institute, Barcelona, Spain; 2 Institut d’Investigacions Biomèdiques August Pi I Sunyer (IDIBAPS), Barcelona, Spain; 3 University of Barcelona, Barcelona, Spain; 4 Centro de Investigación Biomedica En Red- Enfermedades Respiratorias (CIBERES), Barcelona, Spain; 5 University of Nantes, Nantes, France; 6 Department of Infectious Diseases, Beijing Chao-Yang Hospital, Capital Medical University, Beijing, China; 7 Anesthesia and Intensive Care, IRCCS Ospedale Policlinico San Martino, Genoa, Italy; 8 Department of Respiratory and Critical Care Medicine, Beijing Chao-Yang Hospital, Beijing Institute of Respiratory Medicine, Capital Medical University, Beijing, China; 9 Fondazione IRCCS Cà Granda Ospedale Maggiore Policlinico Internal Medicine Department, Respiratory Unit and Adult Cystic Fibrosis Center, and Department of Pathophysiology and Transplantation, Università Degli Studi Di Milano, Milan, Italy; 10 Department of Cardiology, Erasmus MC, University Medical Center Rotterdam, 3015 Rotterdam, The Netherlands; 11 Surgical Intensive Care Unit, Hospital Clínic de Barcelona, Barcelona, Spain; 12 Department of Anesthesiology, Critical Care Medicine and Emergency, SS. Annunziata Hospital, Chieti, Italy; 13 Department of Intensive Care Medicine, Multidisciplinary Intensive Care Research Organization (MICRO), St. James’s Hospital, James’s Street, Dublin, D08 NHY1 Ireland; 14 ISGlobal, Hospital Clínic-Universitat de Barcelona, Barcelona, Spain; 15 Department of Clinical Microbiology, Centre for Biomedical Diagnosis, Hospital Clínic, Barcelona, Spain; 16 Centro de Investigación Biomédica En Red- Enfermedades Infecciosas (CIBERINFEC), Barcelona, Spain; 17 Department of Pathology, Hospital Clinic, Barcelona, Spain; 18 Critical Care Research Group, The Prince Charles Hospital, Chermside, QLD Australia; 19 University of Queensland, St Lucia, QLD Australia; 20 Queensland University of Technology, Kelving Grove, QLD Australia; 21 The Wesley Medical Research, Auchenflower, QLD Australia; 22 Servei de Pneumologia I Al•Lèrgia Respiratoria, Hospital Clínic, Calle Villarroel 170, Esc 6/8 Planta 2, 08036 Barcelona, Spain

**Keywords:** Community-acquired pneumonia, *Streptococcus pneumoniae*, Corticosteroids, Combination antibiotics therapy, Severe pneumonia, Animal model, Mechanical ventilation

## Abstract

**Background:**

*Streptococcus pneumoniae*, a primary cause of community-acquired pneumonia (CAP), is typically treated with β-lactams and macrolides or quinolones. Corticosteroids are now recommended as adjunctive therapy in severe CAP to improve outcomes. **I**n this prospective randomized animal study, we evaluated the bactericidal efficacy of various antibiotic regimens combined with corticosteroids using a porcine pneumococcal pneumonia model.

**Results:**

In 30 White-Landrace female pigs, pneumonia was induced by intrabronchial inoculation of macrolide-resistant *S. pneumoniae* 19A isolate. Animals were randomized to receive saline, ceftriaxone (CRO) with levofloxacin (LVX), CRO with azithromycin (AZM), or combinations of these with methylprednisolone (MP). The primary outcome, *S. pneumoniae* concentrations in lung tissue after 48 h of treatment, showed that the CRO + LVX, CRO + AZM, CRO + LVX + MP, and CRO + AZM + MP groups were equally effective in reducing bacterial load. However, complete bacterial eradication from lung tissue was achieved only in the CRO + AZM + MP group. Secondary outcomes, including bacterial burden in tracheal aspirates and bronchoalveolar lavage (BAL) samples, showed similar bactericidal activity across all treatment groups. The CRO + AZM + MP group demonstrated the most controlled inflammatory response, achieving baseline levels of inflammation, while other groups exhibited elevated inflammatory markers.

**Conclusions:**

Despite using a macrolide-resistant *S. pneumoniae* isolate, the combination of CRO, AZM, and MP achieves similar or even superior results compared to other antibiotic combinations. This regimen provides both bactericidal and immunomodulatory benefits, suggesting its effectiveness in treating macrolide-resistant *S. pneumoniae* pneumonia.

**Supplementary Information:**

The online version contains supplementary material available at 10.1186/s40635-025-00731-1.

## Introduction

*Streptococcus pneumoniae* is one of the most common pathogens responsible for community-acquired pneumonia (CAP), which has a high morbidity and mortality rate (1). Of the anticipated 3.37 million cases annually, approximately 1 million individuals are hospitalized, significantly contributing to the economic burden (2, 3). Among these hospitalized patients, 5% to 10% require admission to the intensive care unit (ICU), and the mortality rate can reach as high as 27% (4).

In the most recent guidelines for severe CAP (sCAP), the panel conditionally recommended—based on very low-quality evidence due to the lack of RCTs—that patients should be initially treated with a beta-lactam plus a macrolide, rather than a fluoroquinolone (5). The combination of these drugs provides extensive coverage against drug-resistant *S. pneumoniae*, although 10 to 15% of *S. pneumoniae* isolates have demonstrated resistance to penicillin and/or macrolide antibiotics (3). The ACCESS trial has also showed that adding clarithromycin to standard-of-care medication enhances early clinical response and attenuates inflammation in hospitalized CAP patients (6). However, the distinct advantages of macrolides over fluoroquinolones, particularly in the context of macrolide resistance, remain to be definitively established (7).

In addition, sCAP patients might present with an excessive inflammatory response, a condition strongly correlated with elevated mortality rates (8). As a potent inhibitor of inflammation, corticosteroids have been suggested for adjunctive use to mitigate cytokine responses (9), shorter time to reach clinical stability (10), and reduced treatment failure (11). Recently, Dequin et al. reported that sCAP patients who received hydrocortisone had a lower risk of death by day 28 than those who received a placebo (12). In this context, current guidelines recommend the use of corticosteroids in sCAP patients (5).

Nonetheless, the precise patient phenotypes that could effectively determine those who would derive the most significant advantages from corticosteroid intervention still need to be fully identified as of current knowledge (5, 13–16). Furthermore, the interaction between corticosteroid and different antibiotic combinations remains poorly understood.

In our previous research, we have developed a novel porcine model of sCAP (17) that allows for a controlled setting where we can explore the detailed interactions between antibiotic combinations and corticosteroids. Hence, we conducted a prospective randomized study in ventilated pigs with severe pneumococcal pneumonia, resistant to macrolides, to assess the effectiveness of corticosteroids plus various antibiotic combinations regarding bactericidal efficacy. Additionally, we evaluated their efficacy on histopathological lung tissue damage, respiratory secretions burden, inflammatory markers, and clinical parameters.

## Material and methods

This study was conducted at the University of Barcelona’s animal facilities in Barcelona, Spain. The study protocol was approved by the Animal Experimentation Ethics Committee of the University of Barcelona (approval reference number: 06/17). All procedures were conducted following the European Directive 2010/63/UE and Spanish RD 53/2013 regulations related to the Guide for the Care and Use of Laboratory Animals and complied with the PREPARE and ARRIVE guidelines.

### Aims of the study

The primary aim was to compare *S. pneumoniae* lung tissue burden in pigs with severe pneumonia undergoing different antibiotic regimens, with or without systemic corticosteroids. Secondary aims include the histological assessment, the evaluation of *S. pneumoniae* concentration in tracheal aspirates and bronchoalveolar lavage (BAL) fluids, the impact on systemic and local inflammation, and the clinical responses.

### Animal preparation and bacterial challenge

For this study, we utilized a recently developed animal model of severe *S. pneumoniae* pneumonia established by our group (17).

Thirty Large White-Landrace female adult pigs (12–16 weeks) were deeply sedated, orotracheally intubated, anesthetized, and mechanically ventilated up to 76 h. Antibiotic prophylaxis with intravenous (IV) doxycycline (100 mg/kg q12 h) and aztreonam (50 mg/kg q8 h) was administered to prevent colonization by endogenous oropharyngeal flora. Using a flexible fiberoptic bronchoscope, a bacterial challenge with 15 mL of 10^8^ CFU/ml of macrolide-resistant *S. pneumoniae* was instilled into both right and left lung pulmonary lobes (Ambu^®^ aScope TM, Columbia, USA). Serotype 19A *S. pneumoniae*—one of the most frequently isolated serotypes in humans—was used (18) and was characterized by resistance to penicillin, macrolides (i.e., MIC > 256 mg/L), and tetracyclines. Twenty-four hours after inoculation, clinical diagnosis of pneumonia was confirmed by the presence in tracheal secretions of ≥ 6 log_10_ CFU/mL of *S. pneumoniae*, plus at least one of the following clinical features: body temperature > 38.5 °C or < 36 °C; white blood count > 14,000/mm^3^ or < 4000/mm^3^ and purulent secretions.

### Randomization

After pneumonia onset, animals were randomized into five groups to receive previously humanized antimicrobial dosage (17):

- 1) IV saline (control, *n* = 6);
- 2) 150 mg/kg IV ceftriaxone + 10 mg/kg IV levofloxacin q24h as 1-h infusion (CRO + LVX, *n* = 6);
- 3) 150 mg/kg IV ceftriaxone + 45 mg/kg IV azithromycin q24h as 1-h infusion (CRO + AZM, *n* = 6);
- 4) 150 mg/kg IV ceftriaxone + 10 mg/kg IV levofloxacin q24h as 1-h infusion + IV bolus of 0.5 mg/kg of methylprednisolone q12h (CRO + LVX + MP, *n* = 6);
- 5) 150 mg/kg IV ceftriaxone + 45 mg/kg IV azithromycin q24h as 1-h infusion + 0.5 mg/kg IV bolus methylprednisolone q12h (CRO + AZM + MP, *n* = 6).

Notably, compared to human dosages, a fourfold increase in the ceftriaxone dose was necessary to achieve a free antibiotic concentration above the *S. pneumoniae* MIC at least 60% of the time (17). Besides, the dosage regimen was confirmed in this study following the same previous methods (Figure S1, Table S1) (17). The corticosteroid dose was used as previously described (11) and given simultaneously with antibiotics.

### Primary outcome

Seventy-six hours after bacterial challenge (i.e., four hours after the last antimicrobial dose), the animals were humanely euthanized by an overdose of anesthetics and a rapid intravenous infusion of 60 mEq KCl. The most affected region of each pulmonary lobe was sampled for microbiological and histological studies (19). The primary outcome was *S. pneumoniae* burden in lung tissue samples. The microbiological cultures were conducted in an experimental microbiological laboratory utilizing previously described methods (17). Quantitative were performed using standard culture methods after aseptically homogenization of 80–120 mg of pulmonary tissue. Samples were serially diluted and plated onto blood and CNA agar. Bacterial counts were calculated as CFU/g after plates incubation during 24–48 h at 37 °C in 5% CO_2_ atmosphere. Bacteria was automatically identified by Microflex LT—MALDI BioTyper 2.0 software (BrukerDaltonics GmbH, Leipzig, Germany) (17). *S. pneumoniae* was also confirmed by optochin susceptibility test (BBL Taxo P disks, BD, Sparks, MD). Pneumonia in each lobe was microbiologically confirmed when a mean *S. pneumoniae* burden ≥ 3 log_10_ CFU/g.

### Secondary outcomes

#### Histopathological examination

Histological damage was blindly assessed in the 149 samples (i.e., five samples per animal except one CRO + LVX animal with four samples; 5 fields for each sample) using two different scoring systems: one for acute lung injury (ALI) following American Thoracic Society (ATS) workshop recommendations (20), and another for pneumonia histological features based on the criteria of Marquette et al. (21). Moreover, the most relevant features for ALI—neutrophils in the alveolar airspaces, septal thickening, airspace filled by proteinaceous debris, and hyaline membranes—were recorded (22).

#### Microbiological assessments

Secondary outcomes in the study included colonization by other pathogens. Also, quantitative microbiology analyses of tracheal secretions, BAL fluids, and blood cultures were performed as previously described for lung tissue (17). Briefly, at baseline and every 24 h thereafter, tracheal secretions and BAL fluids were cultured using standard methods to quantify bacteria and specifically *S. pneumoniae* concentrations. BAL was performed in the right middle lobe using three 10-mL aliquots of sterile saline solution. The first aliquot was discarded, the second was used for quantitative microbiological assessment, and the third was utilized for inflammation analysis.

#### Cytokine analysis

Interferon (INF)-γ; serum interleukin (IL)-1α, IL-R1α, IL-1β, IL-2, IL-4, IL-6, IL-8, IL-10, and IL-18 levels were analyzed in serum and BAL fluids at baseline and every 24 h after bacterial challenge. Both blood and BAL were centrifuge at 3000 rpm at 4 °C for 10 min. Cytokines were quantified by bead-based multiplex assay with Luminex technology for porcine samples(MILLIPLEX^®^ Porcine Cytokine and Chemokine Magnetic Bead Panel, Millipore Iberica, S.A., Madrid, Spain) (17). Cytokine levels in BAL fluids were adjusted using urea as an endogenous marker to obtain ELF concentrations (23). Inflammatory markers data were reported as baseline log_2_ change.

#### Clinical data acquisition and laboratory findings

Pulmonary mechanics were assessed before inoculation and every 24 h. Blood biochemistry was analyzed every 12 h. In addition, ventilatory settings, gas exchange, hemodynamic parameters, and pulmonary variables were recorded and adjusted to maintain clinical stability every 6 h. The vasopressor dependency index (VDI) was calculated by dividing norepinephrine dose (µg/kg/min) by mean arterial pressure (mmHg) and assessed every 6 h.

### Statistical analysis

To ensure the minimal and necessary number of animals required for the study, the sample size was calculated using previous data (17). Based on the expected pulmonary tissue injury scores (i.e., sum of log CFU/g in tissue and Marquette’s score (24))—Control group: 8 points, CRO + LVX: 5 points, CRO + AZM: 4 points, CRO + LVX + MP: 4 points, and CRO + AZM + MP: 3 points—and assuming a standard deviation of 2, a power of 90%, and a type I error rate (α) of 5%, the required sample size was six animals per group.

Continuous variables were reported as median and interquartile range (IQR) [25th–75th percentile], while categorical variables were presented as numbers and percentages. Comparison among groups and over time or pulmonary lobes of continuous variables were assessed using repeated measure two-way ANOVA, followed by post hoc multiple comparisons with false discovery rate (FDR) control. A cumulative link mixed model was used for histological assessment with nested random effects for pigs, lobes, and fields. Tukey's method calculated post hoc pairwise comparisons using estimated marginal means with p-values adjusted for multiple comparisons.

Comparison among groups at fixed time points was assessed using the Kruskal–Wallis non-parametric test, followed by post hoc multiple comparisons controlled by FDR. The Chi-square test was used to compare categorical variables. Significance was considered for *p* ≤ 0.05. All statistical analyses were performed using GraphPad Prism software version 9.0 for Mac (GraphPad Software, San Diego, CA, USA) and R (version 4.3.0).

## Results

Thirty animals (32 [30–33] kg) completed the 76-h study and received an entire course of antibiotics.

### S. pneumoniae lung tissue burden

**Fig. 1:**
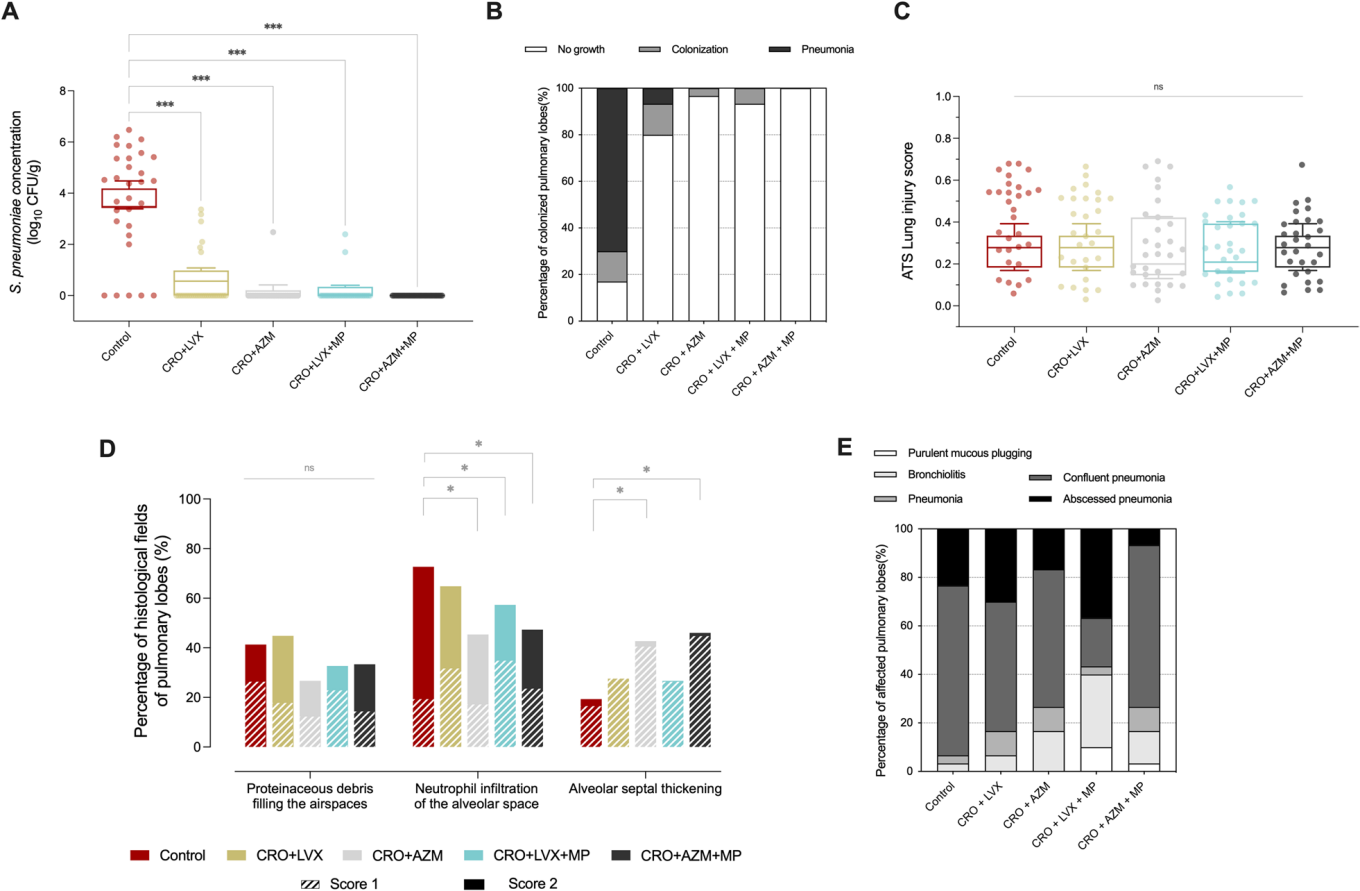
**A** Boxplot displays *S. pneumoniae* concentration (log_10_ CFU/g) in lung tissue among study groups. Horizontal bars represent the median, boxes represent the interquartile range, and whiskers represent the range. Dots represent each of pulmonary lobe burden. There was a notable post hoc FRD-controlled difference in bacterial burden between the control and treatment groups (*p* < 0.0001) but without differences between treated animals. **B** Bars display the percentage of lung tissue concentration of *S. pneumoniae* score among study groups, defined as no growth; *S. pneumoniae* colonization for < 3 log_10_ CFU/g, and pneumonia when *S. pneumoniae* concentration was ≥ 3 log10 CFU/g. The percentage of colonization and pneumonia differed markedly between the control group and treatment groups (*p* < 0.0001), while neither colonization nor pneumonia was found in the CRO + AZM + MP group. **C** Boxplot displays lung injury score following ATS recommendations. No significant differences were observed among study groups (*p* > 0.99).** D** Bars display the percentage of histological features among analyzed microscopic fields (score 1 means 1–5 neutrophils in the alveolar airspaces, twice to four times normal septal or one airspace filled by proteinaceous debris; score two means > 5 neutrophils in the alveolar airspaces, > 4 × septal thickening or > one airspace filled by proteinaceous debris). **E** Bars display the percentage of predominant histological pneumonia features in each pulmonary lobe among study groups. Repeated measures two-way ANOVA with FDR-controlled multiple post hoc comparisons. P-value < 0.05 is flagged with one asterisk, < 0.01 with two asterisks, and < 0.001 with three. *CFU* colony-forming unit, *CRO* ceftriaxone, *LVX* levofloxacin, *AZM* azithromycin, *MP* methylprednisolone

One hundred fifty samples of pulmonary lobes were analyzed (Figure S2). The median [IQR] *S. pneumoniae* tissue concentration was 3.74 [2.92–4.87], 0.29 [0–1.01], 0 [0–0.12], 0 [0-0–38], 0 [0–0] log_10_ CFU/g, in control, CRO + LVX, CRO + AZM, CRO + LVX + MP, CRO + AZM + MP groups, respectively (*p* < 0.001) (Fig. 1A). In post hoc comparisons, bacterial burden was significantly higher in the control group than all other groups. However, no differences were found among animals undergoing any antibiotic treatment, with or without corticosteroids. *S. pneumoniae* was isolated in 25 (83.0%), 6 (20.0%), 1 (3.3%), 2 (6.7%), and 0 (0%) pulmonary lobe tissue samples in the control, CRO + LVX, CRO + AZM, CRO + LVX + MP, and CRO + AZM + MP groups, respectively (p < 0.001) (Fig. 1B).

### Histological findings and colonization by other pathogens

Histopathological analysis of the lung tissue samples is shown in Fig. 1. The ATS histological lung injury score did not differ among study groups (*p* > 0.99). The highest median value was recorded in the control group (0.28 [0.18–0.33]), while the CRO + AZM group presented the lowest (0.19 [0.15–0.42]). However, differences in the histological features of ALI were found among the study groups (Fig. 1D). Indeed, after post hoc analysis, neutrophilic alveolitis was more often found in control animals than in CRO + AZM (*p* = 0.050), CRO + LVX + MP (*p* = 0.012) and CRO + AZM + MP (*p* = 0.042) groups. In contrast, thickened alveolar septae were significantly more frequently found in CRO + AZM (*p* = 0.021) and CRO + AZM + MP (*p* = 0.022) groups than control animals. Of note is that hyaline membranes were not found in any of the fields analyzed across the different study groups.

When samples were analyzed for pneumonia histological features, pneumonia was histologically confirmed in 29 (96.7%), 28 (93.3%), 25 (83.3%), and 25 (83.3%) lung tissue samples in control, CRO + LVX, CRO + AZM, and CRO + AZM + MP groups, respectively, while the lowest figure was found among CRO + LVX + MP (18, 60%), *p* < 0.001 (Fig. 1E). Accordingly, the lung/body weight ratio was 1.71 [1.35–2.47], 1.65 [1.40–1.89], 1.32 [1.29–1.44], 1.22 [1.15–1.28], 1.34 [1.30–1.47] in the control, CRO + LVX, CRO + AZM, CRO + LVX + MP, and CRO + AZM + MP groups (*p* = 0.002), respectively. Significant post hoc differences were observed between CRO + LVX + MP and both the control group (p = 0.002) and the CRO + LVX group (*p* = 0.004), as determined by FRD-controlled analysis (Figure S2).

Although the main isolated pathogen was *S. pneumoniae* in all but one control animal, the lungs were also colonized by other pathogens. These pathogens (i.e., *S. suis, S. aureus, E. faecalis, A. baumannii*) were isolated in 19 (63.3%), 7 (23.3%), 8 (26.7%), 5 (16.7%) and 3 (10%) pulmonary lobes, in the control, CRO + LVX, CRO + AZM, CRO + LVX + MP, and CRO + AZM + MP groups, respectively (p < 0.001) (Figure S3). Accordingly, significant post hoc differences were also observed regarding bacterial burden between control and treated animals.

### Microbiological assessments

**Fig. 2:**
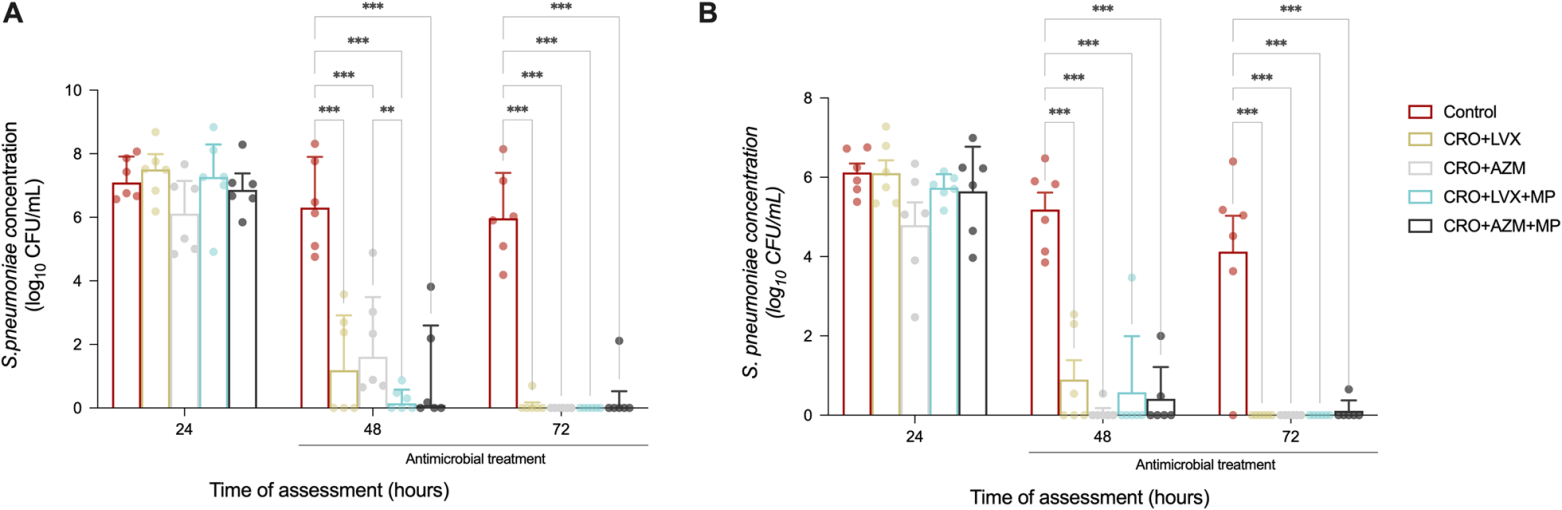
*S. pneumoniae* concentrations (log_10_ CFU/mL) in tracheal secretions (**A**) and bronchoalveolar lavage fluids (**B**) among the study groups. Data are expressed as the mean and standard error of the mean. **A** *S. pneumoniae* concentration in tracheal secretions varied among study groups (p = 0.001) and study time (p < 0.001). Compared with the control group, treatments have shown a crucial impact on the decrease of *S. pneumoniae* colonization in tracheal secretions. At the same time, only statistical significance was reached for CRO + AZM vs CRO + LVX + MP at 48 h among different antimicrobial combinations. **B** Similarly, *S. pneumoniae* concentration in BAL fluid varied among the study groups (p < 0.001) and the study time (p < 0.001). All antimicrobial combinations significantly decrease *S. pneumoniae* BAL burden compared with the control group, control vs. CRO + LVX (p = 0.002), control vs. CRO + AZM (p < 0.001), control vs. CRO + LVX + MP (p = 0.001), control vs. CRO + AZM + MP (p = 0.001). Repeated measures two-way ANOVA with FDR-controlled multiple post hoc comparisons. P-value < 0.05 is flagged with one asterisk, < 0.01 with two asterisks, and < 0.001 with three. *CFU* colony-forming unit, *CRO* ceftriaxone, *LVX* levofloxacin, *AZM* azithromycin, *MP* methylprednisolone

*S. pneumoniae* concentration in tracheal secretion was markedly decreased in treated animals in comparison with the control group (*p* < 0.0001) (Fig. 2A). Moreover, significant post hoc differences were observed between CRO + AZM and CRO + LVX + MP at 48 h (1.61 [0.69–3.48] vs. 0.15 [0–0.58] log CFU/mL, respectively, *p* = 0.006), although both groups reached eradication at the end of the study.

Similarly, the concentration of *S. pneumoniae* in BAL fluid was significantly reduced in the treatment groups compared to the control group throughout the study time (*p* < 0.0001). However, there were no notable post hoc differences in *S. pneumoniae* levels among treatment groups (Fig. 2B). *S. pneumoniae* bacteremia was detected at the end of the study in only one control group subject, while bacteremia was recorded at pneumonia onset in one CRO + LVX animal.

### Inflammatory markers

**Fig. 3:**
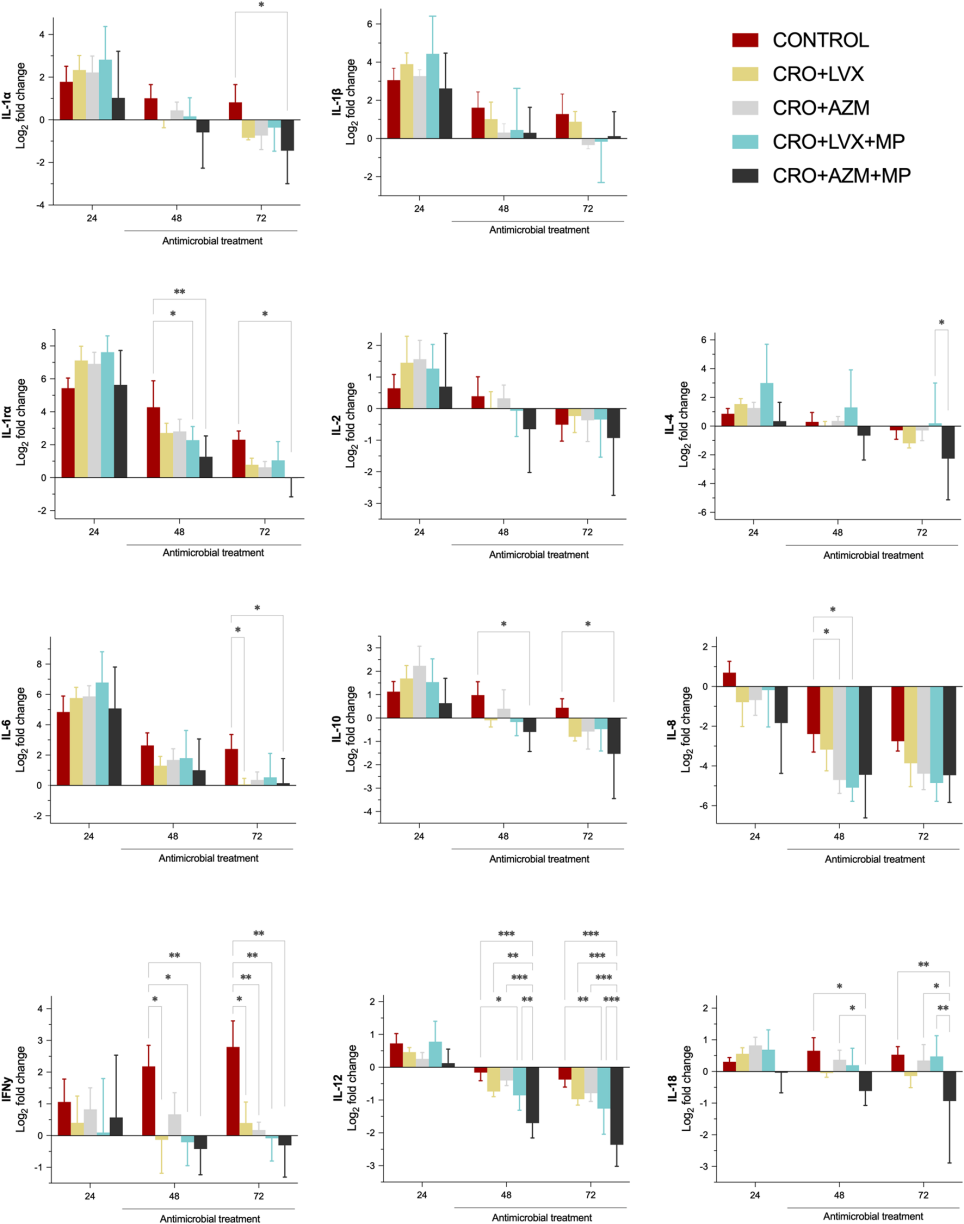
Serum inflammatory markers among study groups. Bars represent the mean fold-change from baseline (log2), and whiskers are the standard deviation. Repeated measures two-way ANOVA without correction for multiple post hoc comparisons. P-value < 0.05 is flagged with one asterisk, < 0.01 with 2 asterisks, and < 0.001 with three asterisks. CRO, ceftriaxone; LVX, levofloxacin; AZM, azithromycin; MP, methylprednisolone

Figure 3 displays the systemic inflammatory markers throughout the study. Although most significant differences were observed in comparison to the control group, the combination of CRO + AZM + MP proved to be the most effective in systemically downregulating both pro-inflammatory and anti-inflammatory cytokines. Significant post hoc differences among treated animals were only observed for systemic IL-4, IL-12, and IL-18, with CRO + AZM + MP outperforming CRO + AZM and CRO + LVX + MP groups.

**Fig. 4:**
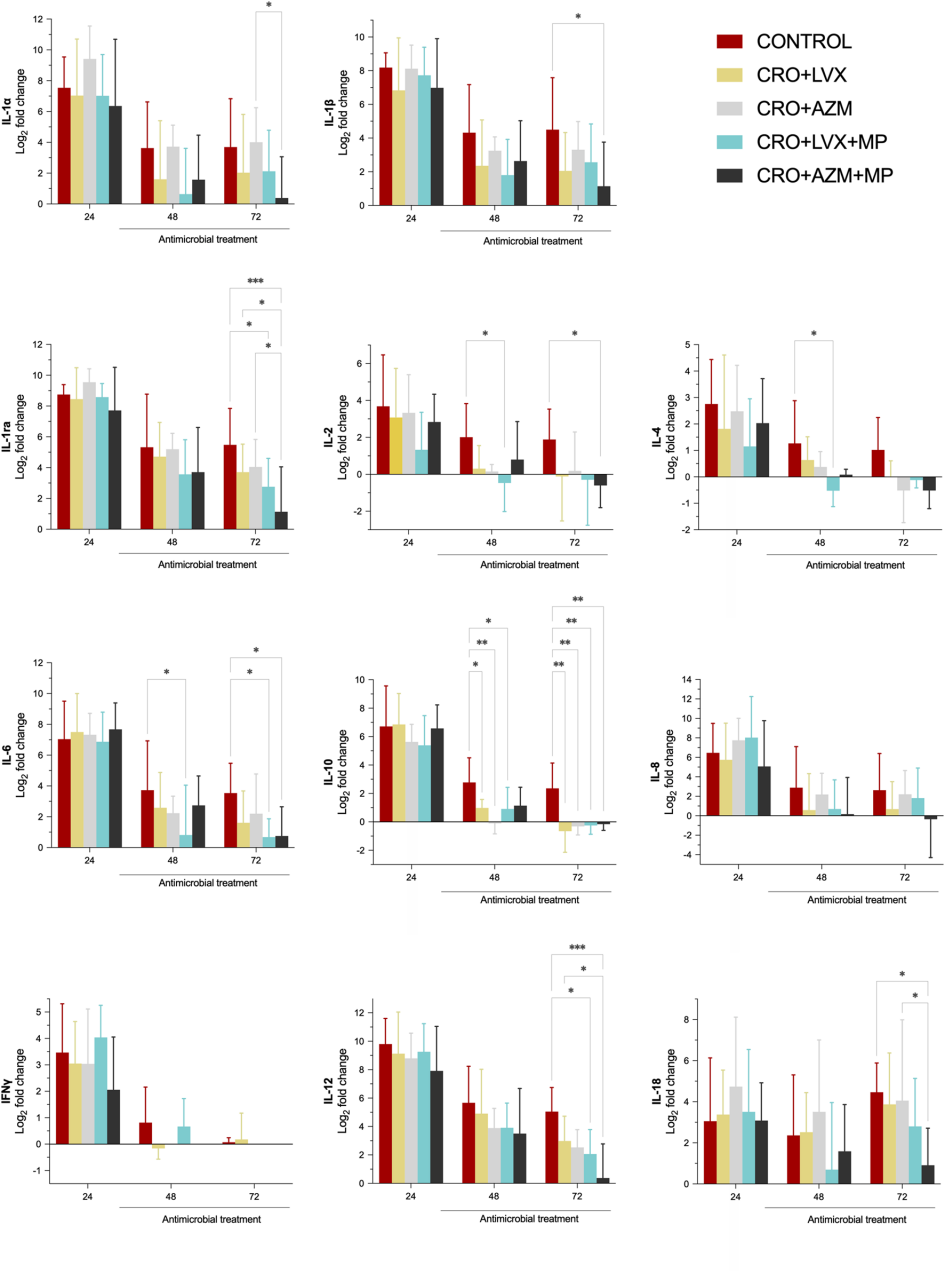
ELF inflammatory markers among study groups. Bars represent the mean fold-change from baseline (log2), and whiskers are the standard deviation. Repeated measures two-way ANOVA without correction for multiple post hoc comparisons. P-value < 0.05 is flagged with one asterisk, < 0.01 with two asterisks, and < 0.001 with three. *CRO* ceftriaxone, *LVX* levofloxacin, *AZM* azithromycin, *MP* methylprednisolone

Similar results were obtained regarding local inflammation (i.e., ELF fluid), with the most notable differences seen when compared to the control group (Fig. 4). Regarding differences between treatments, significant variations in pulmonary IL-12 and IL-18 levels were noted only between CRO + AZM + MP and CRO + LVX and CRO + AZM, respectively, while IL-1Rα was significantly different in both groups in comparison with CRO + AZM + MP.

### Clinical parameters

A comparison among groups regarding respiratory and hemodynamic parameters over time is displayed in Table 1**.** The clinical characteristics were not significantly different at baseline among study groups. Bacterial challenge worsened PaO_2_/FiO_2_, which progressively recovered during treatment. Indeed, 73.3% of the animals met the ARDS criteria (i.e., PaO_2_/FiO_2_ < 300 mmHg) at pneumonia onset (Figure S4). A similar trend was found in respiratory system compliance. Mean pulmonary arterial pressure (mPAP) was increased after bacterial challenge in all groups, but CRO + LVX + MP also attenuated the increase compared to CRO + LVX (*p* = 0.032) treated animals. Vasopressors were required in most of the animals: in all control and CRO + LVX-treated animals and all but one animal in each of the other treatment groups (76.7% at pneumonia onset, Figure S4). However, groups treated with MP (CRO + LVX + MP and CRO + AZM + MP) showed significantly lower VDI compared to the same antibiotic regimens without MP. Specifically, CRO + LVX + MP had lower VDI than the control (*p* = 0.009) and CRO + AZM (*p* = 0.002) groups. Similarly, CRO + AZM + MP showed lower VDI than control (*p* = 0.011) and CRO + AZM (*p* = 0.003). The VDI figures are consistent with the evolution of PaO₂/FiO₂ ratios, mPAP, and pulmonary vascular resistance (PVR), reflecting the interplay between hemodynamic stability, lung perfusion, and oxygenation (Figure S5). Blood biochemistry showed significant changes over the course of the experiment, but no differences were observed among the treatment groups (Table S2).

**Table 1:** Clinical parameters

	Control	CRO + LVX	CRO + AZM	CRO + LVX + MP	CRO + AZM + MP	Time effect	Group effect	Time* group
Respiratory mechanics and ventilator parameters								
C_RS_ (mL/cmH_2_O)								
0 h	28.8 [27.2–30.9]	30 [26.6–31.6]	28.5 [25.5–31.7]	30.3 [27.1–33.3]	27.8 [26–31.7]	** < 0.001**	0.19	**0.008**
24 h	20.8 [19.1–24.6]	19.6 [14.8–22]	19.3 [17.3–21.8]	17.3 [16.5–26.3]	17.7 [15.6–24.4]			
48 h	22.2 [17.6–27]	18.2 [13.8–25.7]	22.5 [19.9–24.9]	25.7 [22.3–33]	24.3 [19.3–27.3]			
72 h	20.3 [16–23.9]	22.2 [15.1–26.1]	25.1 [21.9–34.2]	34.8 [27.4–37.5]	30.1 [24.2–33.3]			
Pulmonary shunt (%)								
0 h	4.4 [3.3–7.4]	6 [4.5–8.4]	6.4 [5.4–9.7]	4.8 [3.3–6.8]	5.5 [3.5–8.2]	** < 0.001**	0.33	0.16
24 h	14.9 [11.8–20.7]	23.3 [15.6–27.7]	14.3 [9.5–25.9]	14.3 [11.3–25.7]	19.3 [11.1–25.3]			
48 h	24.6 [13.5–31.1]	12.1 [8.3–20.8]	14.9 [7.4–23.9]	10 [5.9–22.2]	10.9 [9.2–14.2]			
72 h	15.1 [11.2–19]	14.8 [10.4–20]	11.1 [5.9–17]	7.9 [7–11.2]	14.3 [10.8–18.3]			
MPAP (mmHg)								
0 h	16.5 [14.8–21]	19 [17–20.5]	20 [17.8–21.5]	17 [14–19]	21 [18–22.8]	**0.002**	**0.043**	**0.002**
24 h	24 [18.3–27.8]	28.5 [23.8–29]	25.5 [23.8–28.5]	23 [19.5–26]	26.5 [25–31.5]			
48 h	22.5 [11.5–29.3]	26.5 [22–32]	22.5 [17.5–26.8]	15.5 [13.3–19.3]	21.5 [18–23.3]			
72 h	25 [20.3–33.5]	26 [22–28]	22 [20.3–24]	16.5 [14.8–18]	21.5 [17.5–23.8]			
PPlateau (cmH_2_O)								
0 h	13 [12–13.3]	12.5 [12–13.3]	13.5 [12.8–15.3]	13 [11.8–14.3]	13 [11.8–14]	** < 0.001**	0.55	**0.008**
24 h	21 [20–22.5]	21.5 [20.3–25.8]	24 [21.8—26.5]	24 [20.8–25.5]	23 [21.5–26.8]			
48 h	20 [18.3–25.3]	22.5 [21.3–28.8]	22 [21.8–22.8]	19.5 [18.8–20.5]	19.5 [17.8–22]			
72 h	21.5 [19.8–26]	21 [19–26.5]	20.5 [16.5–22]	17 [15.5–19.5]	18 [17–20.5]			
PEEP (cmH_2_O)								
0 h	4 [4–4.3]	4 [4–4.3]	4 [4–4.3]	4 [4, 5]	4 [4–4]	**0.001**	0.21	**0.005**
24 h	9 [8–10]	9.5 [7.8–10]	10 [10–10]	10 [9.5–10]	10 [9.8–10]			
48 h	8.5 [7.5–9.3]	10 [9.8–10.3]	10 [9.8—10.3]	9.5 [8.8–10]	9 [8.8–10]			
72 h	9 [8–10]	10 [8.8–10.3]	9.5 [8.5–10]	10 [9.8–10]	10 [9, 10]			
FiO_2_ (%)								
0 h	40 [40–40]	40 [40–40]	40 [40–40]	40 [40–40]	40 [40–40]	** < 0.001**	0.92	**0.004**
24 h	65 [53.8–80]	67.5 [55–71.3]	77.5 [75–80]	75 [65–77.5]	75 [65–77.5]			
48 h	57.5 [53.8–76.3]	60 [48.8–81.3]	60 [53.8—67.5]	57.5 [47.5–60]	57.5 [47.5–60]			
72 h	60 [53.8–70]	55 [48.8–76.3]	47.5 [45—55]	52.5 [50–60]	52.5 [50–60]			
Gas exchange								
PaO_2_/FiO_2_								
0 h	455.8 [397.6–505.1]	443.1 [374.8–453.1]	408.6 [352.4–454.1]	414.9 [398.5–444.1]	414.4 [364.5–444.3]	** < 0.001**	0.19	**P = 0.005**
24 h	328.4 [265.2–386.5]	181.5 [153.7–324.2]	314.8 [285.3–370.2]	336.5 [323.5–373.2]	353.1 [302.9–366.8]			
48 h	298.9 [202.3–370.4]	401.1 [249.5–444]	414.7 [349–444.9]	347.4 [219.1–403.8]	334.9 [317.4–365]			
72 h	286 [197.6–386.8]	323.1 [242.3–404.3]	396 [387–446.9]	398.5 [370.9–422.6]	287.7 [253.6–376.5]			
Arterial pH								
0 h	7.51 [7.45–7.54]	7.52 [7.48–7.54]	7.47 [7.46–7.52]	7.47 [7.45–7.48]	7.48 [7.46–7.51]	**0.003**	**0.017**	0.057
24 h	7.43 [7.38–7.46]	7.4 [7.32–7.43]	7.44 [7.38–7.49]	7.47 [7.41–7.5]	7.49 [7.37–7.51]			
48 h	7.41 [7.31–7.48]	7.51 [7.49–7.52]	7.5 [7.47–7.54]	7.48 [7.44–7.56]	7.5 [7.41–7.51]			
72 h	7.45 [7.37–7.46]	7.46 [7.44–7.47]	7.49 [7.48–7.51]	7.5 [7.46–7.51]	7.51 [7.49–7.53]			
Hemodynamic parameters								
VDI								
0 h	0 [0–0]	0 [0–0]	0 [0–0]	0 [0–0]	0 [0–0]	** < 0.001**	**0.005**	** < 0.001**
24 h	0.23 [0–1.12]	0.6 [0.16–2.72]	0.59 [0–1.32]	0.52 [0.18–2.58]	0.57 [0–2.48]			
48 h	2.02 [0.19–4.05]	0.22 [0.11–0.56]	2.18 [0–2.54]	0.04 [0–0.12]	0 [0–0.35]			
72 h	0.22 [0.07–0.57]	0.02 [0–0.1]	0.22 [0–0.79]	0 [0–0]	0 [0–0.01]			
HR (beats/min)								
0 h	86 [67–104]	63 [57.5–97]	87 [77.5–106.25]	72.5 [58.25–96.25]	86.5 [83.5–95]	** < 0.001**	**0.039**	**0.012**
24 h	94.5 [75.5–103]	89.5 [71–104.25]	84.5 [61.5–101]	94.5 [82.5–140.25]	89.5 [69.25–105.75]			
48 h	99 [75.25–182.5]	70 [59–76]	77 [56–94.75]	55 [40–61.75]	58 [55–70.25]			
72 h	73 [58.25–98.75]	52.5 [42.25–59]	52 [48.5–62.75]	46.5 [41.75–51.5]	49.5 [44.25–67.75]			
CO (L/min)								
0 h	3.54 ± 1.00	3.18 ± 0.91	3.82 ± 0.92	3.47 ± 0.87	3.57 ± 0.56	** < 0.001**	0.20	**0.022**
24 h	3.89 ± 1.36	3.94 ± 2.13	4.06 ± 1.79	5.42 ± 2.25	3.85 ± 1.01			
48 h	6.20 ± 2.74	3.39 ± 1.09	4.66 ± 1.44	2.99 ± 0.69	3.30 ± 1.10			
72 h	3.35 ± 1.06	2.58 ± 0.69	3.41 ± 1.41	2.64 ± 0.23	2.64 ± 0.35			

Repeated measures two-way ANOVA with FDR-controlled multiple post hoc comparisons. MPAP differed between CRO + LVX vs. CRO + LVX + MP (*p* = 0.032). Arterial pH differed between control vs. CRO + AZM (*p* = 0.040), CRP + LVX + MP (*p* = 0.005) and CRO + AZM + MP (*p* = 0.005). For VDI, significant differences were observed between CRO + LVX + MP vs. control (*p* = 0.009) and CRO + AZM (*p* = 0.002). Equally, CRO + AZM + MP showed significantly lower VDI than control (p = 0.011) and CRO + AZM (*p* = 0.003) animals. Heart rate was significantly higher in control animals than in CRO + LVX (*p* = 0.009), CRO + LVX + MP (*p* = 0.007), and CRO + AZM + MP- (p = 0.015) treated animals. *AZM* azithromycin, *CRO* ceftriaxone, *CO* cardiac output, *C*_*RS*_ compliance of the respiratory system, *FiO*_*2*_ fraction of inspired oxygen, *HR* heart rate, *LVX* levofloxacin, *MP* methylprednisolone, *MPAP* mean pulmonary arterial pressure, *Pplat* plateau pressure, *PaO*_*2*_*/FiO*_*2*_ arterial partial pressure of oxygen/fraction of inspired oxygen, *PaCO*_*2*_ arterial partial pressure of carbon dioxide, *PEEP* Positive end-expiratory pressure, *VDI* vasopressor index

## Discussion

The main findings of this experimental study in pigs with macrolide-resistant *S. pneumoniae* pneumonia can be summarized as follows: (1) CRO + LVX, CRO + AZM, CRO + LVX + MP, and CRO + AZM + MP had similar bactericidal efficacy in lung tissue, but only in CRO + AZM + MP group *S. pneumoniae was* eradicated in all lung tissue samples; (2) the histological findings showed that pneumonia was present across all groups, with slight improvement of lung/body weight ratio in CRO + LVX + MP; (3) all treatment combinations notably decreased the *S. pneumoniae* concentration in tracheal aspirate and BAL, without a statistically significant difference among treated groups; (4) differences in inflammatory response were mainly observed between control versus treated animals, with slightly better figures in CRO + AZM + MP and CRO + LVX + MP groups; (5) hemodynamics described by VDI were more preserved in animals treated with corticosteroids.

Despite the low resistance rates to macrolides and fluoroquinolones, as reported by the latest European Centre for Disease Prevention and Control (ECDC)—6.5% and 1.1% for *S. pneumoniae,* respectively—higher figures have been observed in Spain and France, with macrolide resistance reaching up to 27%. In this context, no randomized controlled trials (RCTs) have been found that directly compare macrolides to fluoroquinolones in combination with beta-lactam treatment in patients with severe CAP.

Our findings corroborate the efficacy of beta-lactams–macrolide combination, regardless of macrolide resistance. Indeed, Restrepo et al. reported an association between macrolide usage and reduced 30-day and 90-day mortality in severe sepsis due to pneumonia and macrolide-resistant pathogens (25). Similarly, a large, prospective, multicenter study across 27 ICUs in nine European countries found lower ICU mortality associated with macrolides, particularly among more severe cases (26). Some theories may explain the microbiologically pneumonia eradication in the CRO + AZM + MP treated lungs. First, beta-lactam plus macrolide showed better coverage for resistant strains and atypical pathogens (27). Second, β-lactams and macrolides exert a synergistic effect (28, 29); and third, macrolide has an immunomodulatory beneficial impact (30, 31). Considering our serotype of *S. pneumoniae* resistant to macrolides, the immunomodulatory effect of macrolide seems to be the most plausible explanation. Several studies have confirmed that macrolides have immunomodulatory properties, acting on host–pathogen interaction, improving mucociliary clearance, epithelial and inflammatory cells with the final attenuation of the inflammatory response, including many of the primary cytokines, such as IL-1, TNF-α, IL-6, IL-8 (32–34). Macrolides may lead to more efficient pathogen clearance by enhancing the host’s immune response. In our experiment, we were unable to observe a difference between CRO + LVX and CRO + AZM, likely due to a lack of statistical power.

Additionally, routine use of corticosteroids in the clinical setting has been highly debated for several decades. The recent RCT by Dequin et al. demonstrated that sCAP patients experienced a reduced 28-day mortality risk with hydrocortisone treatment starting within 24 h of the onset of sCAP, consistent with our study timeframe (12). Moreover, recent meta-analyses, and current guidelines for sCAP, as well as those for sepsis, acute respiratory distress syndrome (ARDS), and CAP, have reinforced the recommendation of its use in all sCAP patients, regardless of shock (35). However, the interaction of steroids and different antibiotic combinations is still weakly investigated.

Previous studies have shown MP alleviates inflammatory disorders by decreasing the release of cytokines such as IL-1, IL-2, IL-6, IFN-γ, and C-reactive protein; reducing neutrophils, leukocytes, macrophages infiltration to the site of inflammation; and depressing macrophage phagocytosis and accelerated neutrophil apoptosis (36–38). In our study, similar results were observed, with inflammatory markers in serum and ELF (i.e., systemic IL-12 and IL-18, and pulmonary IL-1α, IL-1ra and IL-18 levels) being significantly reduced in the CRO + AZM + MP group compared to the CRO + AZM group. Ceccato et al. previously explored immune modulation in clinical settings (39), and consistent with our recent findings, patients treated with a β-lactam plus macrolide and corticosteroids showed a reduced risk of late treatment failure and an improved biomarker profile, with lower procalcitonin, IL-6, and IL-8 levels on day 3.

A similar trend was observed in LVX groups when MP was added. Specifically, in CRO + LVX with MP group, there was no indication of more severe pneumonia, and the lung/body weight ratio was significantly lower compared to the control group. Previous studies have identified that increasing lung weight was a sensitive biomarker for histopathological changes and inflammatory lesion severity (40, 41). Additionally, systemic and local cytokine levels more often reached baseline levels in the CRO + LVX + MP group than when LVX was administered only in combination with CRO. Notably, significant differences in cytokine levels, particularly in systemic IL-4, IL-12 and IL-18 levels—identified as playing a crucial role in the early antibacterial host response during pneumococcal pneumonia (42)—were observed in the CRO + AZM + MP and CRO + LVX + MP groups compared to the other treatment groups. Also, neutrophilic alveolitis was more often found in control animals than in CRO + AZM, CRO + LVX + MP, and CRO + AZM + MP groups. This superior performance may suggest that this combination is effective in controlling inflammation.

Moreover, the impact of adding MP in our study is also noticed in the VDI with significant post hoc difference between those groups receiving or not corticosteroids treatment. Specifically, the mean pulmonary pressure at the end of the study was significantly lower in CRO + LVX + MP than CRO + LVX. This perhaps accounts for the ability of corticosteroids to modulate pre- and post-capillary blood flow. In fact, this hemodynamic stabilization enhanced pulmonary perfusion, as evidenced not only by reduced PAP but also by decreased PVR and increased PaO₂/FiO₂ ratios. Some studies have also provided evidence that corticosteroids lessen PVR in primary acute respiratory distress syndrome by infectious causes (43). In a *S. pneumoniae* pneumonia mice model, authors found that adding dexamethasone to CRO treatment reduced pulmonary edema, lung permeability and histologic lung injury (44). The reason why the CRO + AZM + MP did not show similar results is unclear and possibly related to the limited power of our post hoc analyses.

This study presents several limitations that deserve further discussion. First, using a porcine model might limit the translatability of our findings into human clinical findings due to the lack of comorbidities and the youth of animals. In control animals, some lobes achieved eradication probably due to potential ununiform application and the healthy status to fight against the pneumonia. However, it is important to acknowledge that conducting an analogous trial in humans presents inherent limitations, primarily due to the impracticality of pre-selecting patients exhibiting resistance to specific antibiotics such as penicillin and AZM. Second, efficacy results and clinical response to treatment were followed up to 48 h post-development of pneumonia, potentially overlooking long-term benefits or harms of the treatments. Third, we used only MP at a single dose based on our prior studies (11, 45), but emerging evidence suggests that hydrocortisone may be more effective for sCAP patients (12, 46, 47). Similarly, the limited sample size did not allow for an analysis of the effect of corticosteroids timing on outcomes. The ceftriaxone dose used was also higher than typical human dosages, likely due to the higher metabolic rate in these animals. Fourth, we used AZM due to its frequent use in clinical practice and based on international guidelines recommendations, but recent studies indicate that clarithromycin, rather than AZM, may confer superior survival benefits in sCAP (6, 48). Lastly, the small sample size and corrections for multiple groups limited the statistical power for some variables.

## Conclusions

Antibiotic combinations and adjunct therapy with corticosteroids demonstrate similar bactericidal efficacy on lung tissue burden in a porcine model of severe macrolide-resistant *S. pneumoniae* pneumonia. Yet, the regimen combining CRO, AZM, and MP exhibits potential superiority in addressing macrolide-resistant *S. pneumoniae* pneumonia. This is especially pertinent in macrolide resistance, offering a synergistic advantage of dual microbiological efficacy and immunomodulatory benefits. Even with the encouraging outcomes, the interpretability is constrained by employing a porcine model and the short study duration.

## Supplementary Information

Supplementary material 1.

## Data Availability

The datasets used and/or analyzed during the current study are available from the corresponding author on reasonable request.

## Abbreviations

ALI: Acute lung injury
ARDS: Acute respiratory distress syndrome
ATS: American Thoracic Society
AZM: Azithromycin
BAL: Bronchoalveolar lavage
CAP: Community-acquired pneumonia
CFU: Colony-forming unit
CRO: Ceftriaxone
ECDC: European Centre for Disease Prevention and Control
ELF: Epithelial lining fluid
FDR: False discovery rate
ICU: Intensive care unit
IL: Interleukin
IQR: Interquartile range
IV: Intravenous
LVX: Levofloxacin
MP: Methylprednisolone
mPAP: Mean pulmonary arterial pressure
PaO2/FiO2: Arterial partial pressure of oxygen/fraction of inspired oxygen
PVR: Pulmonary vascular resistance
RCT: Randomized controlled trial
sCAP: Severe community-acquired pneumonia
VDI: Vasopressor dependency index
