# Supplementary material for: Corticosteroid and antimicrobial therapy in macrolide-resistant pneumococcal pneumonia porcine model
Source: Intensive Care Med Exp. 2025 Feb 27;13:27. doi: 10.1186/s40635-025-00731-1 (PMC11868001; doi:10.1186/s40635-025-00731-1)
Supplement: Supplementary file 1 — Supplementary material 1. [file 40635_2025_731_MOESM1_ESM.docx]

Supplemental Digital Content

**Corticosteroid and Antimicrobial Therapy in Macrolide-Resistant Pneumococcal Pneumonia Porcine Model**

Ana Motos ^1-5^*, Minlan Yang, MD^1-3,6*^, Denise Battaglini^2,7^, Hua Yang^1-3,8^, Andrea Meli^2,9^, Joaquim Bobi^2,3,10^, Roberto Cabrera^1,2^, Giacomo Tanzella^2,7^, Carmen Rosa Vargas^2^, Marta Arrieta^1-3^, Nona Rovira-Ribalta^1,2^, Enric Barbeta^2-4,11^, Pierluigi di Giannatale^2,12^, Stefano Nogas^2,7^, Laia Fernández-Barat^1-4^, Montserrat Rigol^2,3^, Kasra Kiarostami^1-3^, Blanca Llonch^1-3^, Ignacio Martín-Loeches^2,4,13^, Jordi Vila^3,14,15^, Daniel Martinez^3,16^, Gianluigi Li Bassi^17-20^, Antoni Torres, MD, PhD^1-4^

*AM and MY equally contributed.

1. Hospital Clínic, Thorax Institute, Pneumology Department, Barcelona, Spain.
2. Institut d'Investigacions Biomèdiques August Pi i Sunyer (IDIBAPS), Barcelona, Spain.
3. University of Barcelona, Barcelona, Spain.
4. Centro de Investigación Biomedica En Red- Enfermedades Respiratorias (CIBERES), Barcelona, Spain.
5. University of Nantes, Nantes, France
6. Department of Infectious Diseases, Beijing Chao-Yang Hospital, Capital Medical University, Beijing, China.
7. Anesthesia and Intensive Care, IRCCS Ospedale Policlinico San Martino, Genova, Italy
8. Department of Respiratory and Critical Care Medicine, Beijing Chao-Yang Hospital, Capital Medical University, Beijing Institute of Respiratory Medicine, Beijing, China.
9. Fondazione IRCCS Cà Granda Ospedale Maggiore Policlinico Internal Medicine Department, Respiratory Unit and Adult Cystic Fibrosis Center, and Department of Pathophysiology and Transplantation, Università degli Studi di Milano, Milan (Italy)
10. Department of Cardiology, Erasmus MC, University Medical Center Rotterdam, 3015 Rotterdam, The Netherlands.
11. Surgical Intensive Care Unit, Hospital Clínic de Barcelona, Barcelona, Spain.
12. Department of Anesthesiology, Critical Care Medicine and Emergency, SS. Annunziata Hospital, Chieti, Italy.
13. Department of Intensive Care Medicine, St. James's Hospital, Multidisciplinary Intensive Care Research Organization (MICRO), James's Street, D08 NHY1 Dublin, Ireland.
14. ISGlobal, Hospital Clínic-Universitat de Barcelona, Barcelona, Spain; Department of Clinical Microbiology, Centre for Biomedical Diagnosis, Hospital Clínic, Barcelona, Spain.
15. Centro de Investigación Biomédica En Red- Enfermedades Infecciosas (CIBERINFEC), Barcelona, Spain
16. Department of Pathology, Hospital Clinic, Barcelona, Spain
17. Critical Care Research Group, The Prince Charles Hospital, Chermside, QLD, Australia
18. University of Queensland, St Lucia, QLD, Australia
19. Queensland University of Technology, Kelving Grove, QLD, Australia
20. The Wesley Medical Research, Auchenflower, QLD, Australia

**Corresponding author:**

Antoni Torres, MD, PhD

Servei de Pneumologia i Al•lèrgia Respiratòria

Hospital Clínic

Calle Villarroel 170, Esc 6/8 Planta 2

08036 Barcelona (SPAIN)

Voice/Fax: 0034 932275549

Email: atorres@ub.edu

**Table S1. Pharmacokinetic Parameters and Antibiotic Exposure Metrics**

| PIG | CL | V1 | KCP | KPC | Velf | T>MIC plasma | T>MIC elf |
| --- | --- | --- | --- | --- | --- | --- | --- |
| 366 | 5.54 | 7.71 | 0.46 | 1.12 | 7.64 | 56.25 | 52.50 |
| 367 | 10.30 | 19.57 | 5.95 | 4.57 | 99.50 | 88.33 | 63.75 |
| 376 | 5.30 | 4.60 | 0.95 | 0.58 | 10.39 | 77.50 | 82.50 |
| 381 | 4.10 | 3.40 | 0.75 | 0.28 | 11.39 | 100.00 | 100.00 |
| 390 | 7.30 | 5.18 | 1.85 | 0.62 | 10.40 | 81.67 | 97.08 |
| 402 | 5.10 | 19.57 | 0.05 | 0.03 | 34.17 | 100.00 | 97.92 |
| 404 | 6.91 | 9.90 | 3.84 | 4.87 | 14.23 | 65.00 | 59.17 |
| 405 | 7.10 | 8.78 | 0.45 | 0.83 | 9.42 | 59.17 | 58.75 |
| 413 | 9.49 | 3.42 | 4.45 | 0.97 | 31.16 | 59.17 | 56.25 |
| 415 | 6.10 | 8.76 | 0.54 | 1.08 | 8.42 | 60.42 | 58.33 |
| 416 | 12.10 | 13.58 | 1.35 | 1.87 | 21.30 | 48.33 | 43.75 |
| 418 | 3.50 | 8.19 | 0.15 | 0.48 | 5.46 | 89.58 | 89.58 |
| 419 | 7.10 | 8.78 | 0.45 | 0.83 | 9.41 | 57.08 | 56.67 |
| 420 | 9.31 | 3.37 | 9.95 | 1.17 | 74.71 | 85.00 | 72.08 |
| 421 | 6.90 | 9.98 | 3.85 | 4.87 | 14.36 | 62.92 | 57.08 |
| 422 | 7.09 | 8.77 | 0.45 | 0.83 | 9.36 | 55.42 | 55.00 |
| 423 | 5.70 | 7.59 | 0.15 | 1.23 | 2.49 | 42.50 | 40.42 |
| 425 | 7.10 | 8.78 | 0.45 | 0.88 | 14.37 | 56.25 | 50.42 |
| 426 | 9.50 | 1.66 | 5.34 | 0.53 | 39.06 | 68.33 | 66.25 |
| 428 | 9.47 | 1.70 | 5.36 | 0.53 | 39.17 | 68.75 | 66.25 |
| 429 | 19.90 | 2.80 | 9.75 | 0.88 | 79.71 | 47.92 | 43.33 |
| 431 | 7.03 | 8.77 | 0.48 | 0.84 | 9.06 | 57.92 | 58.33 |
| 432 | 9.50 | 1.69 | 5.34 | 0.53 | 38.97 | 69.58 | 67.50 |
| 433 | 6.11 | 8.77 | 0.54 | 1.07 | 8.47 | 60.42 | 58.33 |
| MEDIAN | **7.09** | **8.47** | **0.85** | **0.86** | **12.81** | **61.67** | **58.54** |
| P25 | **6.00** | **3.41** | **0.45** | **0.57** | **9.29** | **56.87** | **55.94** |
| P75 | **9.47** | **8.78** | **4.67** | **1.14** | **35.37** | **78.54** | **68.65** |

CL, clearance; V1, volume of distribution, ; Kcp, rate constant from central to peripheral compartments; Kpc, rate constant from peripheral to central compartments; Velf,Volume of distribution in the epithelial lining fluid; T>MIC plasma, percentage of time the antibiotic concentration exceeds the minimum inhibitory concentration in plasma; T>MIC ELF, percentage of time the antibiotic concentration exceeds the minimum inhibitory concentration in the epithelial lining fluid.

**Table S1.** Blood biochemistry results among the study.

|  | CONTROL | | CRO+LVX | | CRO+AZM | | CRO+LVX+MP | | CRO+AZM+MP | Time effect | Group effect | Time* group |
| --- | --- | --- | --- | --- | --- | --- | --- | --- | --- | --- | --- | --- |
| WBC (10^9^/L) | | |  | |  | |  | |  |  |  |  |
| 0 hrs | 13.65 [9.02 - 15.88] | | 16.31 [9.15 - 28.39] | | 17.05 [9.63 - 22.73] | | 15.85 [13.84 - 18.61] | | 15.27 [12.88 - 16.37] | **<0.001** | 0.36 | **0.003** |
| 24 hrs | 9.91 [7.99 - 22.33] | | 28.1 [12.74 - 55.12] | | 18.88 [11.31 - 25.75] | | 13.63 [9.44 - 24.55] | | 14.19 [7.56 - 29.51] |  |  |  |
| 48 hrs | 24.89 [19.79 - 34.97] | | 32.35 [22.07 - 45.09] | | 30.17 [26.07 - 31.88] | | 39.33 [33.26 - 43.81] | | 43.28 [27.22 - 53.44] |  |  |  |
| 72 hrs | 26.29 [16.15 - 29.32] | | 20.49 [16.33 - 30.87] | | 21.35 [12.37 - 25.25] | | 32.42 [26.03 - 36.09] | | 25.73 [19.11 - 29.37] |  |  |  |
| Creatinine (mg/dL) | |  | |  | |  | |  | |  |  |  |
| 0 hrs | 1.09 [0.94 - 1.22] | | 1.01 [0.88 - 1.15] | | 0.97 [0.9 - 1.19] | | 1.04 [0.97 - 1.2] | | 1.05 [0.99 - 1.12] | 0.30 | 0.16 | 0.16 |
| 24 hrs | 1.11 [0.81 - 1.51] | | 1.19 [1.14 - 1.37] | | 1.15 [0.78 - 1.32] | | 1.08 [0.94 - 1.21] | | 1.03 [0.88 - 1.25] |  |  |  |
| 48 hrs | 1.25 [1.15 - 1.37] | | 1.22 [1.06 - 1.35] | | 1.25 [1.03 - 1.42] | | 0.98 [0.95 - 1.11] | | 0.97 [0.91 - 1.1] |  |  |  |
| 72 hrs | 1.1 [0.95 - 1.28] | | 1.25 [1.03 - 1.48] | | 1.26 [1.03 - 1.49] | | 1.06 [0.86 - 1.08] | | 0.94 [0.79 - 1.12] |  |  |  |
| Platelets (109/L) |  |  | |  | |  | |  | |  |  |  |
| 0 hrs | 587.5 [500.75 - 628] | | 505.5 [420 - 712] | | 550.5 [397.8 - 654.8] | | 470.5 [455.5 - 692.5] | | 516.5 [448.3 - 563.8] | **<0.001** | 0.83 | 0.20 |
| 24 hrs | 393.5 [239 - 457.25] | | 386 [299.5 - 471.8] | | 304 [267.25 - 407.5] | | 331 [277 - 447] | | 317 [275 - 371.5] |  |  |  |
| 48 hrs | 247 [153.25 - 354] | | 268 [159.8 - 290.3] | | 224 [134.5 - 263.25] | | 289 [233.8 - 302.5] | | 253 [221.3 - 346] |  |  |  |
| 72 hrs | 222 [147 - 332] | | 258 [222 - 279.5] | | 183 [156.25 - 247.5] | | 325.5 [231.5 - 342.8] | | 340.5 [232.3 - 465.3] |  |  |  |
| ALT (IU/L) | |  | |  | |  | |  | |  |  |  |
| 0 hrs | 35.5 [20.5 - 54.5] | | 21 [15.5 - 26.5] | | 31.5 [16.75 - 37.25] | | 30 [19.5 - 36.5] | | 30.5 [20.75 - 38] | **0.007** | 0.56 | 0.67 |
| 24 hrs | 21.5 [8 - 40] | | 19 [13.5 - 23] | | 21 [10.5 - 33] | | 21.5 [12.5 - 31] | | 21.5 [17.25 - 29] |  |  |  |
| 48 hrs | 27 [23.75 - 43.75] | | 18 [13.5 - 21] | | 20.5 [7.75 - 36.75] | | 29.5 [12.75 - 41.25] | | 27.5 [20.5 - 31.5] |  |  |  |
| 72 hrs | 40 [16 - 47] | | 22 [13.75 - 28] | | 32.5 [14.25 - 50.5] | | 18.5 [10.25 - 51.25] | | 24.5 [17.25 - 28.25] |  |  |  |
| GGT (IU/L) |  | |  | |  | |  | |  |  |  |  |
| 0 hrs | 69 [43.75 - 95.25] | | 84.5 [70.5 - 124.75] | | 78 [65.5 - 98] | | 78.5 [62.25 - 100] | | 86.5 [65.75 - 98] | **<0.001** | 0.82 | 0.15 |
| 24 hrs | 66 [54.75 - 68.75] | | 70.5 [38.75 - 89.25] | | 57 [46 - 64.5] | | 66 [56 - 96.75] | | 60.5 [36.75 - 70.5] |  |  |  |
| 48 hrs | 63 [46 - 81] | | 51.5 [39.75 - 81.25] | | 55 [30.75 - 73.25] | | 53.5 [46.75 - 61.5] | | 53.5 [41 - 90.75] |  |  |  |
| 72 hrs | 44.5 [42.25 - 50.25] | | 42 [32.25 - 60.5] | | 42 [33.25 - 52.75] | | 58 [55.25 - 64.75] | | 63 [51.75 - 73.75] |  |  |  |
| ALP (IU/L) |  | |  | |  | |  | |  |  |  |  |
| 0 hrs | 159 [103.3 - 223.3] | | 166 [124.8 - 228] | | 140 [107 - 195.5] | | 139 [124.8 - 159.3] | | 140 [115.3 - 173.5] | **<0.001** | 0.68 | 0.086 |
| 24 hrs | 171.5 [112.3 - 194.8] | | 221.5 [179 - 280.5] | | 141 [130.5 - 174.5] | | 198.5 [142 - 234.3] | | 148.5 [123.3 - 210.5] |  |  |  |
| 48 hrs | 153.5 [102.3 - 223] | | 188 [129 - 248.5] | | 171.5 [120.8 - 187.8] | | 180.5 [139.5 - 211.8] | | 157.5 [134.5 - 205] |  |  |  |
| 72 hrs | 120 [74 - 150] | | 116 [85.5 - 144.3] | | 106.5 [103.5 - 122.3] | | 111.5 [96.75 - 124.5] | | 99 [91.25 - 133.25] |  |  |  |
| PT (sec) |  | |  | |  | |  | |  |  |  |  |
| 0 hrs | 10.75 [10.53 - 11.03] | | 10.7 [10.65 - 11.4] | | 11 [10.4 - 11.43] | | 11.7 [10.9 - 13.35] | | 10.85 [10.43 - 11.55] | **<0.001** | 0.68 | 0.064 |
| 24 hrs | 13.5 [11.65 - 14.95] | | 14.45 [14.15 - 15.48] | | 13.6 [13.13 - 14.45] | | 13.35 [12.43 - 14.43] | | 13.85 [12.93 - 14.35] |  |  |  |
| 48 hrs | 13.35 [13.05 - 13.5] | | 13.3 [12.15 - 13.7] | | 13.2 [12.83 - 13.8] | | 12.6 [12.2 - 13.5] | | 12.65 [12.25 - 13.7] |  |  |  |
| 72 hrs | 12.1 [10.98 - 13.4] | | 11.55 [11.08 - 12.8] | | 11.95 [11.53 - 12.33] | | 11.35 [11.08 - 11.58] | | 11.4 [10.2 - 11.9] |  |  |  |
| INR |  |  | |  | |  | |  | |  |  |  |
| 0 hrs | 0.9 [0.89 - 0.96] | | 0.93 [0.91 - 0.97] | | 0.89 [0.88 - 0.95] | | 0.99 [0.89 - 1.16] | | 0.91 [0.89 - 0.95] | **<0.001** | 0.51 | **0.022** |
| 24 hrs | 1.17 [1 - 1.3] | | 1.27 [1.17 - 1.38] | | 1.15 [1.07 - 1.25] | | 1.1 [1.04 - 1.2] | | 1.14 [1.11 - 1.22] |  |  |  |
| 48 hrs | 1.15 [1.09 - 1.18] | | 1.08 [1.05 - 1.18] | | 1.11 [1.07 - 1.15] | | 1.07 [1.02 - 1.11] | | 1.06 [1.04 - 1.14] |  |  |  |
| 72 hrs | 1.02 [0.95 - 1.13] | | 0.96 [0.91 - 1.06] | | 1 [0.95 - 1.02] | | 0.94 [0.89 - 0.96] | | 0.96 [0.87 - 0.97] |  |  |  |

Data are reported as mean ± standard deviation. The p-value of group effect is defined as the probability of differences among five groups during the study. The p-value of time effect is defined as the probability of difference among every 24h of the study from baseline.

ALT, alanine transaminase; ALP, alkaline phosphates; AZM, azithromycin; CRO, ceftriaxone; GGT, gamma-glutamyl transferase; INR, international normalized ratio; LVX, levofloxacin; MP, methylprednisolone; PT, prothrombin time; WBC, white blood cell.

**FIGURE LEGENDS**

**Figure S1. Plasma and epithelial lining fluid pharmacokinetics of ceftriaxone. A)** The upper portion of the figure displays the plasma pharmacokinetics of 150 mg/kg ceftriaxone. **B)** The lower portion of the figure shows the pharmacokinetics in the epithelial lining fluid (ELF). Soft lines depict individual animal data, while the thicker line represents the mean value.

**Figure S2**. **Lungs appearance upon retrieval.** **A)**Weight lung/body percentage among study groups. The mean lung/body weight ratio was 1.71 [1.35 – 2.47], 1.65 [1.40 – 1.89], 1.32 [1.29 – 1.44], 1.22 [1.15 – 1.28], 1.34 [1.30 – 1.47] in the control, CRO+LVX, CRO+AZM, CRO+LVX+MP, and CRO+AZM+MP groups, respectively (p<0.001) with significant post-hoc difference between CRO+LVX+MP vs. control (p=0.002) and CRO+LVX (p=0.004). **B)** Macroscopic signs of pneumonia. Gross signs of pneumonia were found in 26/30 (87%), 22/30 (73%), 20/30 (67%), 21/30 (70%), 25/30 (83%) of the lobes retrieved from control, CRO+LVX, CRO+AZM, CRO+LVX+MPS, and CRO+AZM+MPS groups, respectively (p=0.30). **C)** Pictures of lungs upon autopsy retrieval per study group.

P-value < 0.05 is flagged with one asterisk; < 0.01 with 2 asterisks; and <0.001 with three asterisks. CRO, ceftriaxone; LVX, levofloxacin; AZM, azithromycin; MP, methylprednisolone.

**Figure S3. Other pulmonary lobe colonizers. A)** Bars display percentage of lung tissue concentration by other pathogens among study groups, defined as no growth or colonization when other pathogen is detected. Percentage of colonization differ markedly between control group and treatment groups. (p<0.0001). **B)** Boxplot displays other pathogens concentration (log_10_ CFU/g) in lung tissue among study groups. Horizontal bars represent the median, boxes represent the interquartile range, whiskers represent the range, and plus sign shows the mean. Dots represent each of pulmonary lobe burden. There was notable difference in bacterial burden between control group and treatment groups (p<0.0001) without post-hoc differences between treatment groups.

P-value < 0.05 is flagged with one asterisk; < 0.01 with 2 asterisks; and <0.001 with three asterisks. CRO, ceftriaxone; LVX, levofloxacin; AZM, azithromycin; MP, methylprednisolone.

**Figure S4. ARDS criteria and vasopressor use across treatment groups and timepoints. A)** Percentage of ARDS criteria; bars represent the proportion of animals meeting ARDS criteria at different timepoints. **B)** Percentage of animals on vasopressors (%), bars show the percentage of animals requiring vasopressors over time across the groups.

ARDS, Acute Respiratory Distress Syndrome; CRO, ceftriaxone; LVX, levofloxacin; AZM, azithromycin; MP, methylprednisolone.

**Figure S5. Interplay among hemodynamics, lung perfusion and oxygenation variables.** **A)** PaO2/FiO2 values displayed over time in hours for each treatment group. B) Mean pulmonary arterial pressure displayed over the course of 72 hours. **C)** Pulmonary vascular resistance over 72 hours **D)** Vasopressor dependency index reveals significant trends and differences in vasopressor use across groups.

CRO, ceftriaxone; LVX, levofloxacin; AZM, azithromycin; MP, methylprednisolone; PaO2/FiO2, Arterial Partial Pressure of Oxygen/Fraction of Inspired Oxygen.

**Figure S1**

**Figure S2**

**Figure S3**

**Figure S4**

**Figure S5**
